# Establishing an intravenous sotalol loading program

**DOI:** 10.1016/j.hroo.2024.11.011

**Published:** 2024-11-22

**Authors:** Keturah DelGrosso, Kathryn Wood

**Affiliations:** 1 Geisinger, Enterprise Pharmacy, Danville, Pennsylvania; 2 Emory University, Nell Hodgson Woodruff School of Nursing, Atlanta, Georgia

**Keywords:** Sotalol/administration and dosage, Antiarrhythmic drugs/therapeutic use, Administration, intravenous, atrial fibrillation

## Abstract

Atrial fibrillation (AF) is the most frequent cardiac arrhythmia seen clinically with serious sequelae of stroke and heart failure as well as an independent risk factor for increased mortality. The latest guidelines for AF management recommend restoration and maintenance of normal sinus rhythm through the use of antiarrhythmic drugs, cardioversion, or catheter ablation treatment early after AF diagnosis to lower the risk of adverse cardiovascular outcomes. Intravenous (IV) sotalol loading for atrial arrhythmias allows for rapid achievement of the maximum plasma concentration steady state, where patients receive a 60-minute infusion, followed by 2 oral doses of the drug. Practical questions remain about establishing an IV sotalol program. While IV sotalol is not a new medication, the expanded indication for use of a loading dose requires pharmacy and therapeutics committee review of published data, such as indication, dosage, plans for patient and corrected QT monitoring, electrolyte monitoring and replacement, if necessary, order set and protocol development, and a timeline for the infusion process. The purpose of this article is to describe development of an IV sotalol loading program that addresses formulary approval, order set and protocol tips, and preadmission and admission considerations, including monitoring corrected QT intervals, staffing, and locations for the IV sotalol infusion process.

**Key Findings**

▪ Rapid intravenous (IV) sotalol loading over 60 minutes for atrial arrhythmias achieves the maximum plasma concentration (Cmax) steady state in <24 hours in patients with creatinine clearance > 60 mL/min as compared with ≥72 hours with oral sotalol loading, resulting in a decreased length of stay.
▪ Publications regarding IV sotalol’s cost-effectiveness, efficacy, and safety are important to provide to the pharmacy and therapeutics committee to receive formulary approval.
▪ Steps to develop an IV sotalol program include formulary approval, development of protocol, order set, and infusion process timeline, as well as preadmission and discharge process strategies.

## Introduction

Recent research and atrial fibrillation (AF) management guidelines recommend restoration and maintenance of normal sinus rhythm through the use of antiarrhythmic drugs or other treatments early after AF diagnosis.^1^, ^2^, ^3^, ^4^ Sotalol is a Vaughan-Williams class III antiarrhythmic drug approved in 1992 as an oral medication for maintenance of normal sinus rhythm in patients with atrial arrythmias and life-threatening ventricular arrythmias.^5^^,^^6^ While sotalol is an effective drug, it brings significant concerns related to corrected QT (QTc) prolongation.^7^ Hospitalization for a minimum of 3 days with electrocardiographic (ECG) monitoring is required for oral sotalol loading.^8^ In patients with creatinine clearance (CrCl) > 60 mL/min, oral sotalol loading requires 5 doses to reach the maximum plasma concentration (Cmax) steady state.^8^^,^^9^

In March 2020, intravenous (IV) sotalol received an expanded indication as a loading dose for atrial arrythmias where patients receive a 60-minute infusion, followed by 2 oral doses of sotalol.^8^, ^9^, ^10^, ^11^, ^12^, ^13^ The extent of QTc prolongation is seen earlier with IV loading, as the Cmax steady state is rapidly achieved in <24 hours in patients with CrCl > 60 mL/min as compared with 3 days for traditional oral sotalol loading.^10^^,^^14^ This shortened length of stay improves patient satisfaction and overall cost savings.^15^

Information about the process of IV sotalol loading in AF has been limited. Much of the available published data refer to statistical modeling of simulation data.^16^ We will describe strategies for implementing a 1-day IV sotalol loading program.

## Formulary approval

While IV sotalol is not a new medication, the expanded indication requires pharmacy and therapeutics committee review of published data, such as indication, dosage, and plans for patient and QTc monitoring.^11^, ^12^, ^13^, ^14^, ^15^^,^^17^ The differences between oral and IV sotalol loading should be highlighted, specifically the same safety and efficacy, cost-effectiveness data, and reduced length of stay with the IV formulation.^12^ While the cost of oral sotalol tablets is significantly less than that of an IV sotalol vial, it is important to include the total cost of admission and length of stay. Varela et al^15^ evaluated the cost of oral vs IV sotalol loading in 133 patients with AF and projected cost savings of ∼$3803 per admission for 1-day IV sotalol loading as compared with 3-day oral loading. In another study, Lakkireddy et al^11^ reported that IV sotalol loading (60-minute infusion), followed by 1 oral dose, and then discharging the patient 4 hours after the first oral dose was safe and effective in patients with AF. In addition, a potential cost savings of ∼$3500 per patient admission (which could be translated to $350,068 per 100 patients when comparing 1-day IV sotalol loading with 3-day oral loading) was reported.^11^

## Protocol and order set development

Once approved on the formulary, protocol and order set development should begin. The protocol should address electrolyte replacement, place of infusion, and QTc monitoring and measurement. The Food and Drug Administration (FDA) suggests normalizing both potassium and magnesium before the infusion, as IV sotalol is contraindicated with a potassium of <4 mEq/L.^8^ Institutions should decide not only on thresholds to initiate electrolyte replacement for both potassium and magnesium, but also on how to replace it (ie, oral vs IV replacement and dosing).

When deciding on the location of the IV sotalol infusion, available bed space, staff, and cost should be considered. Per the FDA prescribing directions, IV sotalol must be infused in a location with continuous ECG monitoring and cardiac resuscitation equipment readily available.^8^ Options for infusion may include telemetry units, cardiac units, infusion centers, and the emergency department. While intensive care units may have more nursing resources, the cost of using a bed in this location may outweigh the benefit.

Finally, the protocol should outline how QTc interval will be monitored and measured. Per the FDA, the QTc interval must be monitored every 15 minutes during the infusion, but it does not specify how to monitor the QTc interval.^8^ Some institutions may choose to complete four 12-lead ECGs, while others may review telemetry strips. The protocol should provide instructions as to who is reviewing the QTc interval and when the provider should be contacted. If the QTc interval prolongs >450 ms during the admission, the protocol should outline appropriate actions such as terminating the sotalol infusion and notifying the provider.

Order set development should align with the protocol to ensure safe and effective administration of IV sotalol. Laboratory orders (metabolic panel, magnesium, complete blood count, international normalized ratio, etc), electrolyte replacement orders, IV sotalol dosing (Table 1), oral sotalol dosing, ECG orders, nursing orders, and discharge orders should be included. The IV sotalol loading dose is based on target oral dose and CrCl (Table 1). For ease of computerized order entry, consider a drop-down menu with correct IV dose based on these 2 factors. The order set should also appropriately time the first and second oral doses, depending on renal function and ending time of the IV sotalol infusion. Nursing orders should include how often to take vital signs and when to contact the physician if the QTc interval prolongs during the admission, as well as frequency and timing for ECG monitoring. Commonly, a baseline ECG is completed, followed by ECGs 2 hours after each sotalol dose.

**Table 1:** Intravenous (IV) sotalol loading dose recommendations

Creatinine clearance∗	To initiate IV sotalol loading for the target of 80 mg oral dose, the IV loading dose to be administered over 1 h is:	To initiate IV sotalol loading for the target of 120 mg oral dose, the IV loading dose to be administered over 1 h is:	If needed to increase sotalol dosing from 80 to 120 mg oral dose, the IV loading dose to be administered over 1 h is:	If needed to increase sotalol dosing from 120 to 160 mg oral dose, the IV loading dose to be administered over 1 h is:	The minimum time to wait before delivery of the first oral dose	Oral dosing interval is every:
>90 mL/min	60 mg	90 mg	75 mg	90 mg	4 h	12 h
60–90 mL/min	82.5 mg	125 mg	82.5 mg	105 mg	4 h	12 h
30–60 mL/min	75 mg	112.5 mg	82.5 mg	105 mg	6 h	24 h
10–30 mL/min	75 mg	112.5 mg	82.5 mg	105 mg	12 h	48 h

∗ Creatinine clearance calculated using the Cockcroft-Gault formula.

The overall timeline of the infusion process is often overlooked. For example, if a patient with CrCl > 60 mL/min undergoes IV sotalol loading from 0800 to 0900, the first oral dose would occur at 1300, followed by the second oral dose at 0100. This is not a convenient outpatient dosing schedule for most patients, and institutions should consider starting the infusion at a time that will allow the oral doses to be administered during waking hours.

## Preadmission considerations

When a patient is identified to undergo IV sotalol loading, there are several outpatient considerations to ensure success. First, find an IV sotalol loading “champion” within the physician’s office. The champion will facilitate the logistical steps and considerations for successful IV sotalol loading. The champion should coordinate with the hospital admissions staff to schedule the patient for IV sotalol loading. Second, the champion should review admission instructions with the patient such as time of arrival to hospital, any pre-hospitalization fasting instructions and instructions about admission laboratory tests and ECG, and medications.

Patients should be informed of any laboratory work and ECGs needed before admission. Commonly, preadmission laboratory work such as metabolic panel, magnesium, and complete blood count are completed within 3–7 days before the IV sotalol load. This is to ensure that patients have adequate renal function to continue with the planned IV sotalol loading as well as provide an opportunity for electrolyte replacement. Replacing and normalizing potassium and magnesium before admission may help to avoid any delays during IV sotalol loading. In addition, an ECG before admission (within 30 days) to evaluate baseline rhythm and QTc interval should be considered. If a patient presents in AF or atrial flutter, consideration for cardioversion in addition to IV sotalol loading may be needed. Patients also undergoing cardioversion should be compliant with anticoagulation before admission. For patients on warfarin, the office champion should ensure that the international normalized ratio laboratory draws are scheduled at the correct frequency before admission and the provider should review results to ensure that readings are therapeutic. Finally, the patient should be told whether they take all their home medications before admission. Atrioventricular nodal blockers are often held before admission to reduce the risk of bradycardia during the sotalol loading process.

## Admission process

**Figure 1:**
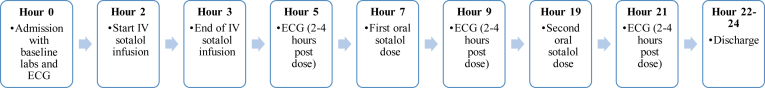
Example timeline for 24-hour IV sotalol loading in patients with creatinine clearance > 60 mL/min. ECG = electrocardiogram; IV = intravenous; labs = laboratory tests.

When patients arrive for IV sotalol loading and possible cardioversion, an observation status vs admission may be considered in patients with CrCl > 60 mL/min discharged within < 24 hours. Figure 1 depicts a successful <24 hours IV sotalol loading timeline to allow patient and institutional satisfaction. One study found that the mean length of stay in 95 patients receiving IV sotalol loading was 1 day as compared with 4.1 days with oral sotalol loading.^12^ Another study observed the mode length of stay for IV sotalol loading was 24 hours as compared with 102 hours in patients receiving oral sotalol loading.^15^ While patients with CrCl 30–60 mL/min will have a longer stay, IV sotalol loading can still be completed in <48 hours (less than traditional oral sotalol loading that commonly takes ≥72 hours).

## Discharge and follow-up

After successful sotalol initiation, providing patients with oral sotalol in hand at discharge may help improve compliance and eliminate missing dose issues due to delays in obtaining sotalol from personal pharmacies. This may be particularly important if the patient is discharged in the evening when some pharmacies are closed. Furthermore, patients should have appropriate follow-up scheduled before discharge.

## Conclusion

IV sotalol loading is an important option for rhythm control in patients with atrial arrythmias. Because of its ability to reach the Cmax steady state in <24 hours, length of stay is significantly reduced in comparison to oral sotalol loading, resulting in increased patient satisfaction as well as cost savings for both the patient and the hospital. To successfully use IV sotalol loading within an institution, it is important to prepare for pharmacy and therapeutics committee approval, create a protocol and order set, and appropriately identify IV sotalol loading candidates.
